# A uracil auxotroph *Toxoplasma gondii* exerting immunomodulation to inhibit breast cancer growth and metastasis

**DOI:** 10.1186/s13071-021-05032-6

**Published:** 2021-12-11

**Authors:** Li-Qing Xu, Li-Jie Yao, Dan Jiang, Li-Juan Zhou, Min Chen, Wen-Zhong Liao, Wei-Hao Zou, Hong-Juan Peng

**Affiliations:** Department of Pathogen Biology, Guangdong Provincial Key Laboratory of Tropical Disease Research, School of Public Health, Southern Medical University, Guangzhou, Guangdong 510515 People’s Republic of China

**Keywords:** *Toxoplasma gondii*, Uracil auxotroph, In situ inoculation, Breast cancer, Immunomodulation

## Abstract

**Background:**

Breast cancer is the most common cause of cancer-related death among women, and prognosis is especially poor for patients with triple-negative breast cancer (TNBC); therefore, there is an urgent need for new effective therapies. Recent studies have demonstrated that the uracil auxotroph *Toxoplasma gondii* vaccine displays anti-tumor effects. Here, we examined the immunotherapy effects of an attenuated uracil auxotroph strain of *T. gondii* against 4T1 murine breast cancer.

**Methods:**

We constructed a uracil auxotroph *T. gondii* RH strain via orotidine 5′-monophosphate decarboxylase gene deletion (RH-Δ*ompdc*) with CRISPR/Cas9 technology. The strain’s virulence in the *T. gondii*-infected mice was determined in vitro and in vivo by parasite replication assay, plaque assay, parasite burden detection in mice peritoneal fluids and survival analysis. The immunomodulation ability of the strain was evaluated by cytokine detection. Its anti-tumor effect was evaluated after its in situ inoculation into 4T1 tumors in a mouse model; the tumor volume was measured, and the 4T1 lung metastasis was detected by hematoxylin and eosin and Ki67 antibody staining, and the cytokine levels were measured by an enzyme-linked immunosorbent assay.

**Results:**

The RH-Δ*ompdc* strain proliferated normally when supplemented with uracil, but it was unable to propagate without the addition of uracil and in vivo, which suggested that it was avirulent to the hosts. This mutant showed vaccine characteristics that could induce intense immune responses both in vitro and in vivo by significantly boosting the expression of inflammatory cytokines. Inoculation of RH-Δ*ompdc *in situ into the 4T1 tumor inhibited tumor growth, reduced lung metastasis, promoted the survival of the tumor-bearing mice and increased the secretion of Th1 cytokines, including interleukin-12 (IL-12) and interferon-γ (INF-δ), in both the serum and tumor microenvironment (TME).

**Conclusion:**

Inoculation of the uracil auxotroph RH-Δ*ompdc* directly into the 4T1 tumor stimulated anti-infection and anti-tumor immunity in mice, and resulted in inhibition of tumor growth and metastasis, promotion of the survival of the tumor-bearing mice and increased secretion of IL-12 and IFN-γ in both the serum and TME. Our findings suggest that the immunomodulation caused by RH-Δ*ompdc* could be a potential anti-tumor strategy.

**Graphical abstract:**

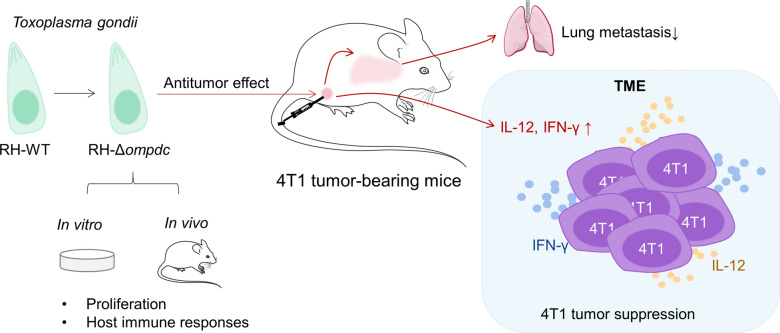

**Supplementary Information:**

The online version contains supplementary material available at 10.1186/s13071-021-05032-6.

## Background

Breast cancer is the most common malignancy and the most frequent cause of cancer-related death in women worldwide, with a global incidence of 24.2% and a mortality of 15% [1]. Triple-negative breast cancer (TNBC) is defined as a breast tumor lacking the expression of estrogen receptor (ER), progesterone receptor (PR), and human epidermal growth factor receptor-2 (HER2). Among the subtypes of breast cancer, TNBC is the most aggressive form of breast cancer, with the highest recurrence rate and poorest prognosis [2–4]. Nowadays, surgery, radiation therapy and chemotherapy are the main therapeutic options for the treatment of breast cancer in clinical practice, and chemotherapy remains the mainstay of TNBC treatment [2, 5, 6]. Although a large number of novel therapies have been evaluated, the effects of clinical treatment on TNBC remain poor [7], which emphasizes the need to explore new therapies for effective TNBC treatment.

The tumor microenvironment (TME) is a complex cellular environment composed of multiple cells that affects tumor progression [8]. In an immunosuppressive TME, various immunosuppressive cells, such as T regulatory cells (Tregs), myeloid-derived suppressor cells (MDSCs), tumor-associated macrophages (TAMs) and type II natural killer T cells (NKTIIs), contribute to immunosuppression through various mechanisms that promote cancer progression [8]. Cancer immunotherapy can reverse an immunosuppressive TME and remodel the immune system to kill tumor cells [9, 10]. Various immunosuppressive mechanisms are present in the developmental process of mammary glands [11–13], and these may be utilized by breast cancer cells to influence the development and progression of breast cancer according to immune surveillance and immune editing principles [14–16]. In the early phase, the immune microenvironment mostly exerts anti-tumor effects, inhibiting tumor progression by producing cytokines and activating natural killer (NK), CD4^+^ and CD8^+^ T cells [2], suggesting that immunotherapy is a promising strategy to treat breast cancer. Some immunotherapy approaches are effective; for example, the immune checkpoint inhibitor pembrolizumab (targeting programmed cell death protein 1 [PD-1]) is clinically efficacious in metastatic TNBC patients with PD-L1^+^ tumors, with a reported overall response rate of 18.5% [17]. However, many effective immunotherapy strategies are often associated with serious complications; for instance, paients treated with immune checkpoint inhibitors have many adverse events, such as cardiovascular toxicity, which has a relatively high mortality rate [18]. Therefore, safe and effective therapeutic strategies are urgently needed.

Microorganisms have long been considered potential immunotherapy agents against cancers. In previous studies, many organisms have shown prominent anti-tumor effects in various cancer models. These include *Listeria monocytogenes*, *Salmonella typhimurium* and many other programmable bacteria [19–25], the oncolytic virus *Talimogene laherparepvec* [26], cowpea mosaic virus [27–30], yellow fever vaccine 17D [31] and the artificial attenuated parasite *Toxoplasma gondii* [32–37], all of which have been reported to be able to inhibit the growth or metastasis of tumors as well as improve prognosis. Genetic manipulation enables the generation of avirulent or attenuated microorganisms that can induce powerful immune responses, making them effective immune-stimulating anti-tumor agents.

*Toxoplasma gondii* is an obligate intracellular parasite that can induce immune responses defined by the production of interleukin-12 (IL-12) and interferon-γ (IFN-γ) [38] that are similar to the host immune responses against breast cancer. An analysis of the changes in tumor-related factors following *T. gondii* infection revealed that this bacterium affects the breast cancer signaling pathway [39], leading us to conjecture whether the progression of breast cancer could be inhibted by infection with a *T. gondii* vaccine. Non-replicating avirulent uracil auxotroph vaccine strains (such as Δ*cps* and Δ*ompdc*) have been reported to grow normally in a culture medium supplemented with exogenous uracil, but be unable to replicate in mice [40, 41]. Earlier studies reported that a Δ*cps* vaccine could inhibit the growth of some solid tumors, including mouse Lewis lung carcinoma [33], B16F10 melanoma [34], ID8-*Defb29/Vegf-A* ovarian cancer [35] and Pan02 pancreatic cancer [36, 37]. A Δ*cps* vaccine was found to be able to reverse the immunosuppression of tumors, increase the production of type 1 helper cell (Th1) cytokines IL-12 and IFN-γ and activate tumor-related CD8^+^ T cells to recognize and kill tumor cells [32–37]. These results suggest that the avirulent *T. gondii* strain may be an effective immunotherapeutic agent for TNBC treatment.

In our study, we constructed an avirulent uracil auxotroph RH-Δ*ompdc* strain that could grow normally in mammalian cells when supplemented with uracil, but which was unable to propagate without uracil and in mice. The potential of the avirulent RH-Δ*ompdc* strain as a biotherapy agent in 4T1 tumor treatment was also evaluated.

## Methods

### Animal and animal ethics

BALB/c mice were obtained from the Animal Experimental Center of Southern Medical University. All animal experimental procedures were approved by the Experimental Animal Care and Use Committee of Southern Medical University (permit no. L2019155). All mice were housed under standard conditions with unlimited access to food and water.

### Cell lines

Human foreskin fibroblast (HFF), murine RAW264.7 cells and 4T1 cells were purchased from ATCC (American Type Culture Collection, Manassas, VA, USA) and incubated at 37 °C under 5% CO_2_. HFF and RAW264.7 cells were cultured in DMEM (Dulbecco’s Modified Eagle Medium; Gibco/Invitrogen, Thermo Fisher Scientific, Waltham, MA, USA), and 4T1 cells were cultured in RPMI-1640 (Roswell Park Memorial Institute-1640; Gibco/Invitrogen) supplemented with 10% (v/v) fetal bovine serum (FBS), 100 units/ml of penicillin (Thermo Fisher Scientific) and 100 μg/ml of streptomycin (Thermo Fisher Scientific).

### Parasite culture

*Toxoplasma gondii* tachyzoites were maintained in HFF monolayers cultured in DMEM supplemented with 1% FBS (v/v). The uracil auxotroph RH-Δ*ompdc* strain was additionally supplied with 0.2 mM uracil (Sigma-Aldrich, St. Louis, MO, USA) [40, 41]. Freshly egressed parasites purified by passage through a 3-μm polycarbonate filter (Whatman plc, GE Healthcare, Chicago, IL, USA) were used in all experiments.

### Generation of *T. gondii* OMPDC gene knockout strain

The orotidine 5′-monophosphate decarboxylase (OMPDC) gene knockout strain (RH-Δ*ompdc*) was constructed with CRISPR/Cas9 technology based on an reciprocal hemizygous wild-type strain (RH-WT) following a published protocol [42]. Gene-specific CRISPR plasmid and homologous template were co-transfected into RH-WT tachyzoites. The RH-Δ*ompdc* strain was cultured in HFF cells and screened in the medium with 0.2 mM uracil and 3 μM pyrimethamine (Sigma-Aldrich) by limiting dilution. Positive clones were identified by PCR and quantitative reverse transcription PCR (qRT-PCR) using the primers listed in Additional file 2: Table S1.

### Parasite replication assay and plaque assay

*Toxoplasma gondii* intracellular proliferation was observed using an indirect immunofluorescence assay. HFF cells were grown on coverslips to 100% confluence and then infected with tachyzoites of the RH-WT or RH-Δ*ompdc* strain at a multiplicity of infection of 1. The infected cells were cultured in a medium supplemented with or without 0.2 mM uracil for 24 h, following which the cells were fixed with methanol, incubated with mouse anti-SAG1 monoclonal antibody (Abcam, Oxford, UK) and incubated with an Alexa Fluor 594-conjugated goat anti-mouse IgG (Invitrogen) according to the manufacturer’s guidance for visualizing parasites. The number of the parasitophorous vacuoles (PVs) containing 1, 2, 4, 8 or 16 parasites at different replication stages over 100 vacuoles was counted independently from three separate coverslips. The experiment was repeated three times for statistical analysis.

For the plaque assay, HFF monolayers were grown on coverslips in a six-well plate to 100% confluence, and then infected with 1000 tachyzoites per well of of the RH-WT or RH-Δ*ompdc* strain. These infected cells were cultured with or without 0.2 mM uracil for 6 days, following which the cells were fixed with 4% (v/v) paraformaldehyde and stained with 0.1% (v/v) crystal violet; then the number and size of the plaques were analyzed [43].

### RNA extraction and qRT-PCR

RAW264.7 cells were infected with RH-WT and RH-Δ*ompdc* tachyzoites without uracil supplement for 24 h, following which total RNA of the *T. gondii* tachyzoites and RAW264.7 cells was extracted using a TRIzol reagent (Invitrogen) and reversely transcribed to cDNA with one-step gDNA removal by using TransScript® II All-in-One First-Strand cDNA Synthesis SuperMix (TransGen Biotech, Beijing, China), following the manufacturer’s instructions. The transcription levels of IL-12, IL-6, IL-1β, inducible nitric oxide synthase (iNOS) and tumor necrosis factor-α (TNF-α) normalized with glyceraldehyde 3-phosphate dehydrogenase (GAPDH) were examined by qRT-PCR using a Hieff™ qPCR SYBR® Green Master Mix (Low Rox; Yesen, China). The relative mRNA levels were calculated with the comparative ΔCt method using the formula 2^−ΔΔCt^. All qRT-PCR reactions were performed in technical triplicates. The primers are listed in Additional file 2: Table S2.

### Evaluation of the virulence and protective efficacy of RH-Δ*ompdc*

For virulence detection, the freshly egressed RH-WT and RH-Δ*ompdc* tachyzoites were purified as mentioned above and resuspended in phosphate-buffered saline (PBS). The 6- to 8-week-old BALB/c mice were infected with 100 tachyzoites of the RH-WT strain or 10^6^ or 2 × 10^6^ tachyzoites of the RH-Δ*ompdc* strain by intraperitoneal (i.p.) injection (8 mice per group). The survival of mice was observed for 30 days.

For protective efficacy evaluation, the mice were immunized with PBS or with 10^6^ or 2 × 10^6^ RH-Δ*ompdc* tachyzoites per mouse for 30 days (7 mice per group). The mice were then challenged with 100 RH-WT tachyzoites per mouse, and the mice were observed for 30 days.

For the determination of the parasite burden in mice peritoneal fluids, the peritoneal fluids were collected from the infected mice at different time points (12 h, 24 h and 5 days) after infection. Genomic DNA was extracted using the DNeasy Blood & Tissue Kit (Qiagen, Hilden, Germany). The parasite burden in the peritoneal fluids was determined by detecting the B1 gene copies with qPCR (for primers, see Additional file 2: Table S1). Quantitative PCR was performed with the Hieff™ qPCR SYBR® Green Master Mix (Low Rox) following the manufacturer’s instructions. The standard curve for B1 gene copies (indicating the number of *T. gondii*) was generated as previously described [44].

### Construction of mouse tumor model and treatment with RH-Δ*ompdc*

The 4T1 cells were harvested, washed twice with RPMI-1640 medium and resuspended in PBS at a concentration of 10^6^ cells/ml. The right flank of 5- to 6-week-old female BALB/c mice was subcutaneously (s.c.) injected with 100 µl of the mixture. Tumors were allowed to grow for 8 days until their sizes reached 30–50 mm^3^. The tachyzoites of RH-Δ*ompdc* were harvested and resuspended in PBS to a concentration of 10^7^ ml^−1^. PBS and RH-Δ*ompdc* were slowly intratumorally (i.t.) injected at one site using a 26 G injection needle with an injection volume of 100 µl to the control and experimental group, respectively. The tumor-bearing mice were treated every 7 days for a total of four treatments. The tumor size was measured every 2 days using vernier calipers and calculated following the formula: Volume (V) = 0.52 × (length × width^2^) [45].

### Cytokine assay

The following enzyme-linked immunosorbent (ELISA) kits were used to detect the cytokines based on the manufacturer’s recommendations: Mouse IL-12p40 ELISA Kit (EK2183/2) and Mouse IFN-γ ELISA Kit (EK280/3) (MultiSciences, Hangzhou, Multisciences (Lianke) Biotech, Co., Ltd., China). Normal mice were infected with RH-WT or RH-Δ*ompdc* tachyzoites, and tumor-bearing mice were treated with PBS or RH-Δ*ompdc* tachyzoites, as described above. Tumors were harvested at 1, 8, and 13 days after the first treatment and then sliced and homogenized at 4 °C; serum from each mouse was collected accordingly. The tumor samples were centrifuged at 5000 *g* for 5 min, and serum samples were centrifuged at 1000  *g* for 15 min. The supernatants were subsequently used to measure IL-12 and IFN-γ levels using the ELISA kits.

### Analysis of lung metastasis for 4T1 tumor

On day 13 after the first treatment, the mice treated with PBS and RH-Δ*ompdc* (*n* = 3) were euthanized, and the lungs were harvested and fixed in 4% (v/v) paraformaldehyde for 2 days. The fixed tissues were then sent to the Servicebio Biotechnology Company (Wuhan, China) for paraffin embedding, slicing and subsequent staining of the slices with hematoxylin/eosin (H&E). For immunohistochemistry, the slices were stained with the Ki67 antibody followed by the goat anti-rabbit HRP secondary antibody (Servicebio Biotechnology Company). The stained slices were visualized and photographed with a Nikon microscope using a 20× 0.75 NA objective (Nikon Corp., Tokyo, Japan). The number of positive cells per square millimeter was automatically calculated with Image-Pro Plus 6.0 software.

### Statistical analysis

Data were analyzed and charts were generated using GraphPad Prism 6 (GraphPad Software Inc., San Diego, CA, USA) . A two-tailed Student’s t-test was used to compare differences between groups. Both one-way and two-way analysis of variance with Tukey’s multiple comparisons test were used to compare differences among multiple groups. Survival data were analyzed using the log-rank (Mantel–Cox) test. Statistical significance was defined as *P* < 0.05.

## Results

### The OMPDC gene was essential to the proliferation and growth of *T. gondii *in vitro

The defective mutants of de novo pyrimidine biosynthesis are uracil auxotrophs, which are nonreplicating and avirulent in mice [40, 41]. In order to study the therapeutic effects of the nonreplicating *T. gondii* on 4T1 mouse breast cancer, we constructed the uracil auxotrophic mutant with CRISPR/Cas9 technology. The construction schematic diagram is shown in Additional file 1: Figure S1a. The PCR and qRT-PCR results demonstrated that the RH-Δ*ompdc* mutant of *T. gondii* was successfully constructed (Additional file 1: Figure S1b, c).

**Fig. 1:**
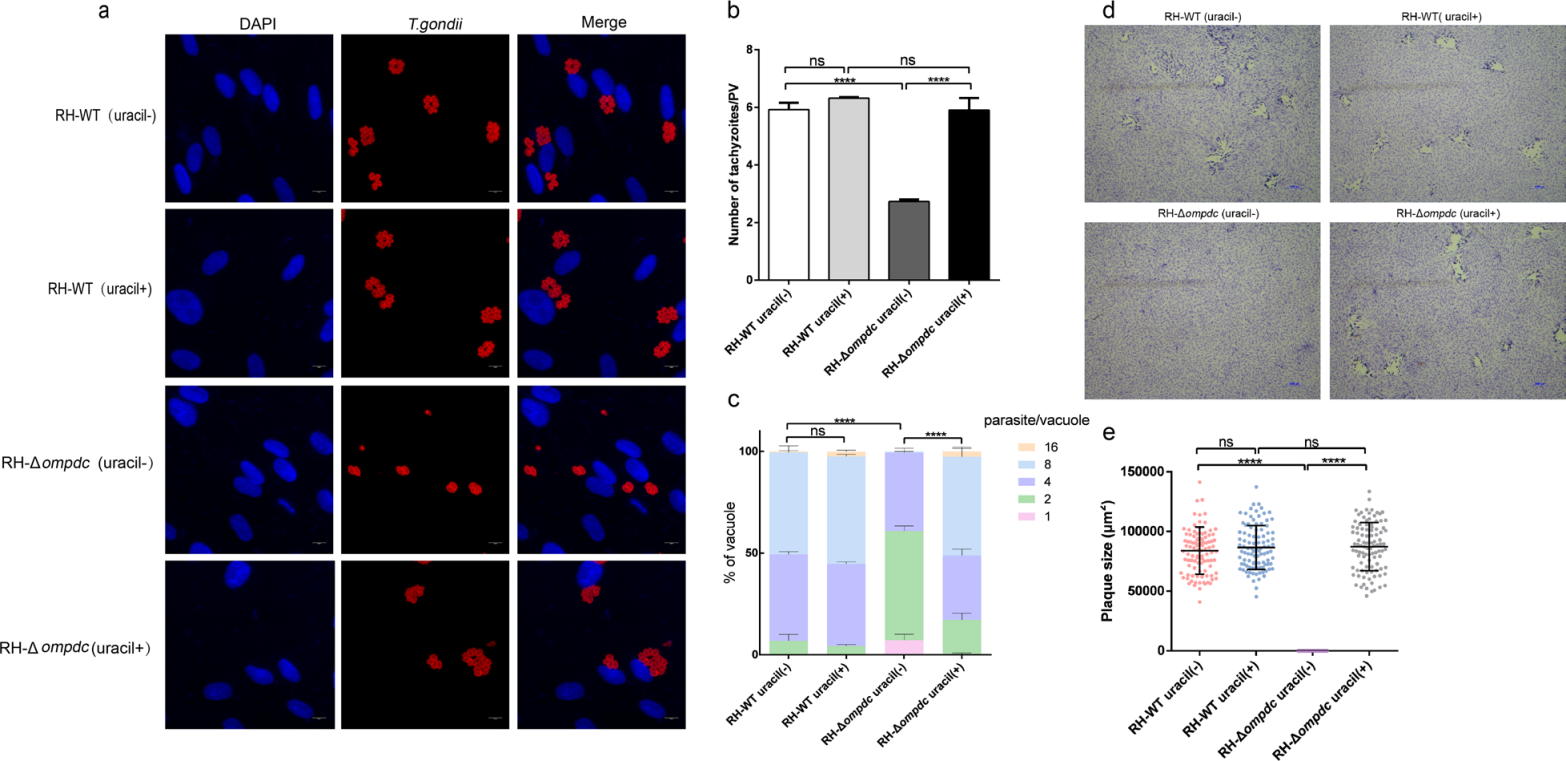
The OMPDC gene was essential to the proliferation of *Toxoplasma gondii *in vitro. **a**–**c** The detection of *T. gondii* proliferation in the indicated groups by an immunofluorescence assay at 24 h post infection: **a** HFF cells infected with the indicated tachyzoites with or without uracil supplement were observed under a fluorescence microscope, **b** the number of tachyzoites per PV was counted, **c** percentages of PVs containing different numbers (1, 2, 4, 8 or 16) of the indicated *T. gondii* strains. **d** Proliferation of the RH-WT and RH-Δ*ompdc* strains was compared with plaque assay. **e** Areas (μm^2^) of the plaques formed by the RH-WT and RH-Δ*ompdc* strains supplemented with or without uracil. Results are representative of three independent experiments. Data were analyzed by ANOVA (**b**, **e**) and by two-way ANOVA (**c**) with Tukey’s test. Asterisks indicate significant difference at ****P < 0.0001; ns, not significant. Abbreviations: ANOVA, Analysis of variance; RH-Δ*ompdc*, OMPDC gene knockout strain; PV, parasitophorous vacuole; RH-WT, reciprocal hemizygous wild-type strain

Parasite replication and plaque assays were used to examine the effects of the OMPDC gene on the intracellular proliferation of *T. gondii*. The parasite replication assay showed that at 24 h post infection, the number of RH-WT tachyzoites per PV was significantly higher than that of the RH-Δ*ompdc* mutant in the absence of uracil. The number of tachyzoites per PV of the RH-Δ*ompdc* mutant supplied with uracil was remarkably higher than that supplied without uracil, although uracil addition not did not affect the proliferation of the RH-WT strain (Fig. 1a–c). These results indicated that the proliferation of the RH-Δ*ompdc* mutant was significantly inhibited in the absence of uracil, and that uracil addition could rescue the reduced proliferation phenotype of the RH-Δ*ompdc* mutant. Plaque assay results showed us that the RH-Δ*ompdc* auxotroph could grow normally in the presence of uracil to form plaques, but it failed to do so in the absence of uracil. However, the RH-WT strain was able to grow normally in the culture medium with or without uracil (Fig. 1d, e). These results led us to conclude that the OMPDC gene was essential to the proliferation and growth of *T. gondii *in vitro and that the replication of the RH-Δ*ompdc* mutant could be recovered by uracil supplement.

### RH-Δ*ompdc* infection significantly increased the transcription levels of proinflammatory cytokines in vitro

**Fig. 2:**
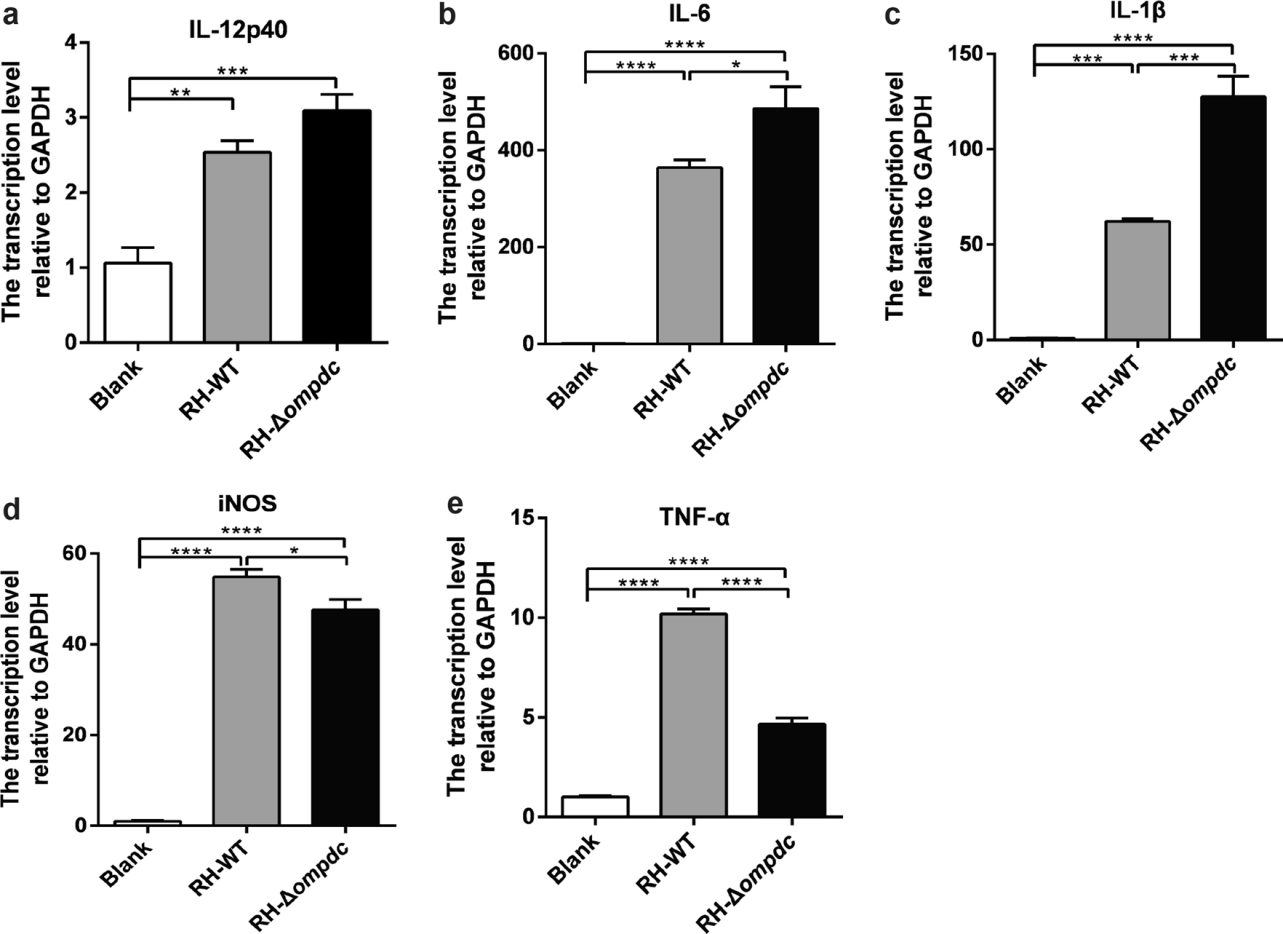
Relative transcription levels of cytokines in RAW264.7 cells after RH-WT and RH-Δ*ompdc* infection. The relative transcription levels of IL-12p40 (**a**), IL-6 (**b**), IL-1β (**c**), iNOS (**d**) and TNF-α (**e**) normalized with GAPDH were significantly increased in both RH-WT and RH-Δ*ompdc* infection groups compared to the control group. Results are representative of three independent experiments. Data were analyzed by one-way ANOVA with Tukey’s test. Asterisks indicate significant difference at *P < 0.05, **P < 0.01, ***P < 0.001 and ****P < 0.0001.

Proinflammatory cytokines, which are predominantly produced by activated immune cells, play central roles in inflammatory diseases. To investigate the ability of the RH-Δ*ompdc* auxotroph to activate macrophages and induce host immune responses, we measured the transcription levels of the cytokines in RAW264.7 cells in the absence of uracil. RAW264.7 cells were infected with RH-WT and RH-Δ*ompdc* tachyzoites without uracil supplement for 24 h, following which the total RNA was extracted and the mRNA levels of cytokines were detected by qRT-PCR. The relative transcription levels of IL-12, IL-6, IL-1β, iNOS and TNF-α were significantly increased after *T. gondii* infection in RAW264.7 cells, regardless of OMPDC gene deletion (Fig. 2). These results indicated that RH-Δ*ompdc* infection significantly increased the transcription levels of proinflammatory cytokines and may induce strong immune responses in hosts.

### Vaccination with the avirulent RH-Δ*ompdc* protected the mice from infection by virulent RH strain

**Fig. 3:**
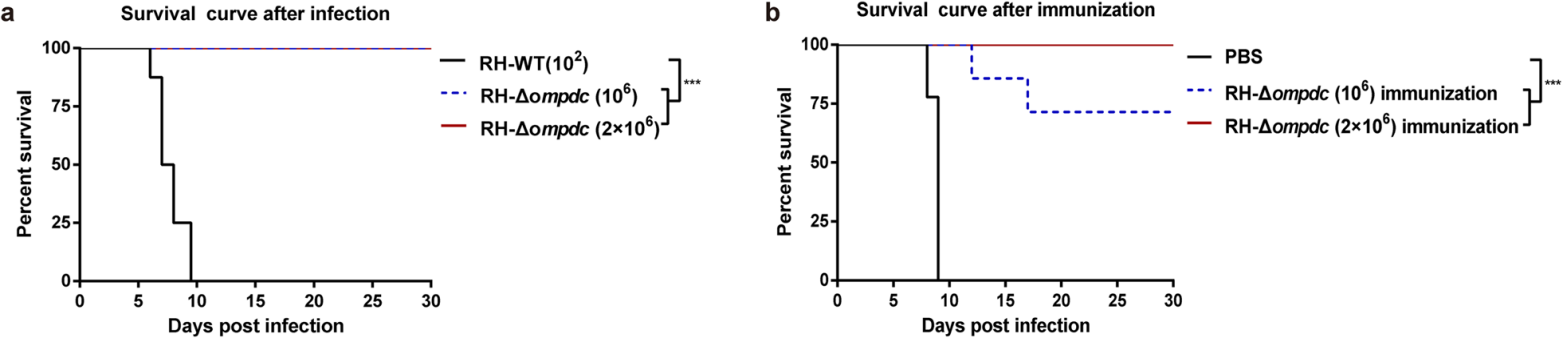
Detection of the virulence and protective effects of the RH-Δ*ompdc* strain in mice. **a** Attenuated virulence of the RH-Δ*ompdc* strain in mice was observed. Mice (*n* = 8) infected with 100 RH-WT tachyzoites (i.p.) all died within 10 days, and mice (*n* = 8) infected with 10^6^ or 2 × 10^6^ RH-Δ*ompdc* tachyzoites (i.p.) all survived. **b** RH-Δ*ompdc* immunization provided protection against RH-WT infection. After being immunized with PBS or 1 × 10^6^ or 2 × 10^6^ RH-Δ*ompdc* (i.p.) for 30 days, the mice were challenged with 100 RH-WT tachyzoites (i.p.). The mice in the PBS group (*n* = 7) all died within 9 days after the challenge, while the mice in the 1 × 10^6^ and 2 × 10^6^ RH-Δ*ompdc* immunization groups (*n* = 7) showed survival rates of 71.4 and 100%, respectively. Results are representative of three independent experiments. Data were analyzed by survival curves: log-rank (Mantel–Cox) test. Asterisks indicate significant difference at ***P < 0.001. Abbreviations: i.p., intraperitoneal injection; PBS, phosphate-buffered saline

To estimate the virulence of the RH-Δ*ompdc* mutant in vivo, BALB/c mice were inoculated i.p. with the RH-WT and RH-Δ*ompdc* tachyzoites. Those mice infected with 100 RH-WT tachyzoites each died within 10 days, while all mice infected with 10^6^ or 2 × 10^6^ RH-Δ*ompdc* tachyzoites survived (Fig. 3a). These results indicated that the virulence of the RH-Δ*ompdc* mutant was severely attenuated. The protective effects of the RH-Δ*ompdc* immunization on mice against its parental RH strain infection were further assessed. Mice were immunized (i.p.) with PBS or with 10^6^ or 2 × 10^6^ RH-Δ*ompdc* tachyzoites for 30 days and then challenged with 100 RH-WT tachyzoites. At the end of the 30-day observation period, the survival rate of the mice vaccinated with 10^6^ RH-Δ*ompdc* tachyzoites was 71.4% and that of the mice vaccinated with 2 × 10^6^ tachyzoites was 100% (Fig. 3b). These results demonstrated that RH-Δ*ompdc* was avirulent in mice and that vaccination with this mutant could protect mice from reinfection by its parental virulent RH strain.

### RH-Δ*ompdc* could not propagate in mice but could induce intense immune responses during acute infection

**Fig. 4:**
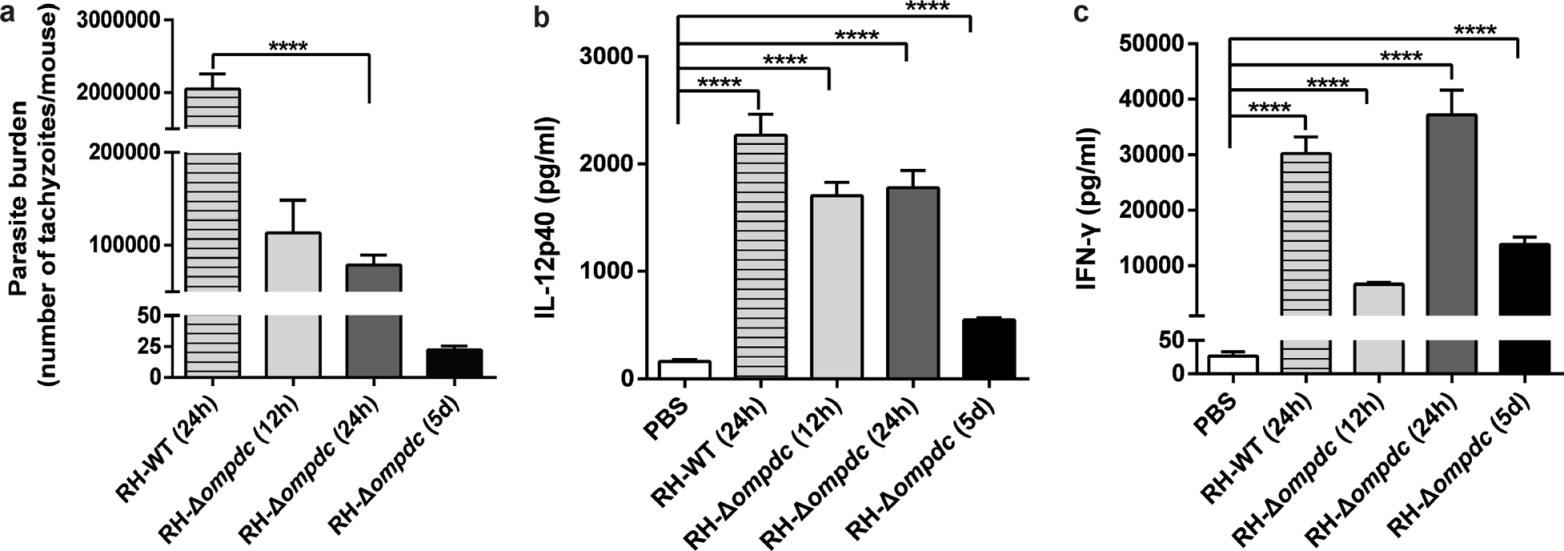
The RH-Δ*ompdc* mutant could be eliminated in mice and induced intense host immune responses. The 6- to 8-week-old BALB/c mice were infected with 10^6^ RH-WT and RH-Δ*ompdc* tachyzoites (i.p.), and the parasite burden in the peritoneal fluids and the levels of IL-12 and IFN-γ in the serum were detected at 0, 12, 24 h, and 5 d post infection. **a** The parasite burden in the peritoneal fluids of mice (*n* = 4) infected with RH-WT and RH-Δ*ompdc* tachyzoites was detected at the indicated time post infection. The B1 gene was detected by qPCR to demonstrate the number of *T. gondii* tachyzoites in each sample. **b** IL-12 level in mice serum (*n* = 5). **c** IFN-γ level in mice serum (*n* = 4). Results are representative of three independent experiments. Data were analyzed by one-way ANOVA with Tukey’s test. Asterisks indicate significant difference at ****P < 0.0001

The proliferative ability of RH-Δ*ompdc* in mice and the levels of the cytokines IL-12 and IFN-γ in mice serum were further assessed. Mice were infected with 10^6^ tachyzoites of RH-WT and RH-Δ*ompdc* by i.p. injection, and the parasite burden in the peritoneal fluids was subsequently examined by qPCR at 12 h, 24 h and 5 days after infection. At 24 h post infection, RH-WT displayed robust replication and high parasite burden in the peritoneal fluids, while RH-Δ*ompdc* showed a significant reduction in intraperitoneal parasites, and was eliminated within 5 days after infection (Fig. 4a). The mice sera were collected at different time points after infection for the detection of the Th1 cytokines IL-12 and IFN-γ. The levels of IL-12 and IFN-γ in the serum were significantly upregulated after RH-WT and RH-Δ*ompdc* infection (Fig. 4b, c). This result indicated that RH-Δ*ompdc* induced strong host immune responses, similarly to its parental RH strain. However, in the RH-Δ*ompdc* infection group, the levels of IL-12 and IFN-γ first increased and then rapidly declined although they were still remarkably higher than those in the control group at 5 days past infection (Fig. 4b, c). In brief, RH-Δ*ompdc* could be rapidly cleared in mice but also induced a strong immune responses, as with its parental RH strain.

### The highly attenuated *T. gondii* strain RH-Δ*ompdc* inhibited 4T1 tumor growth

**Fig. 5:**
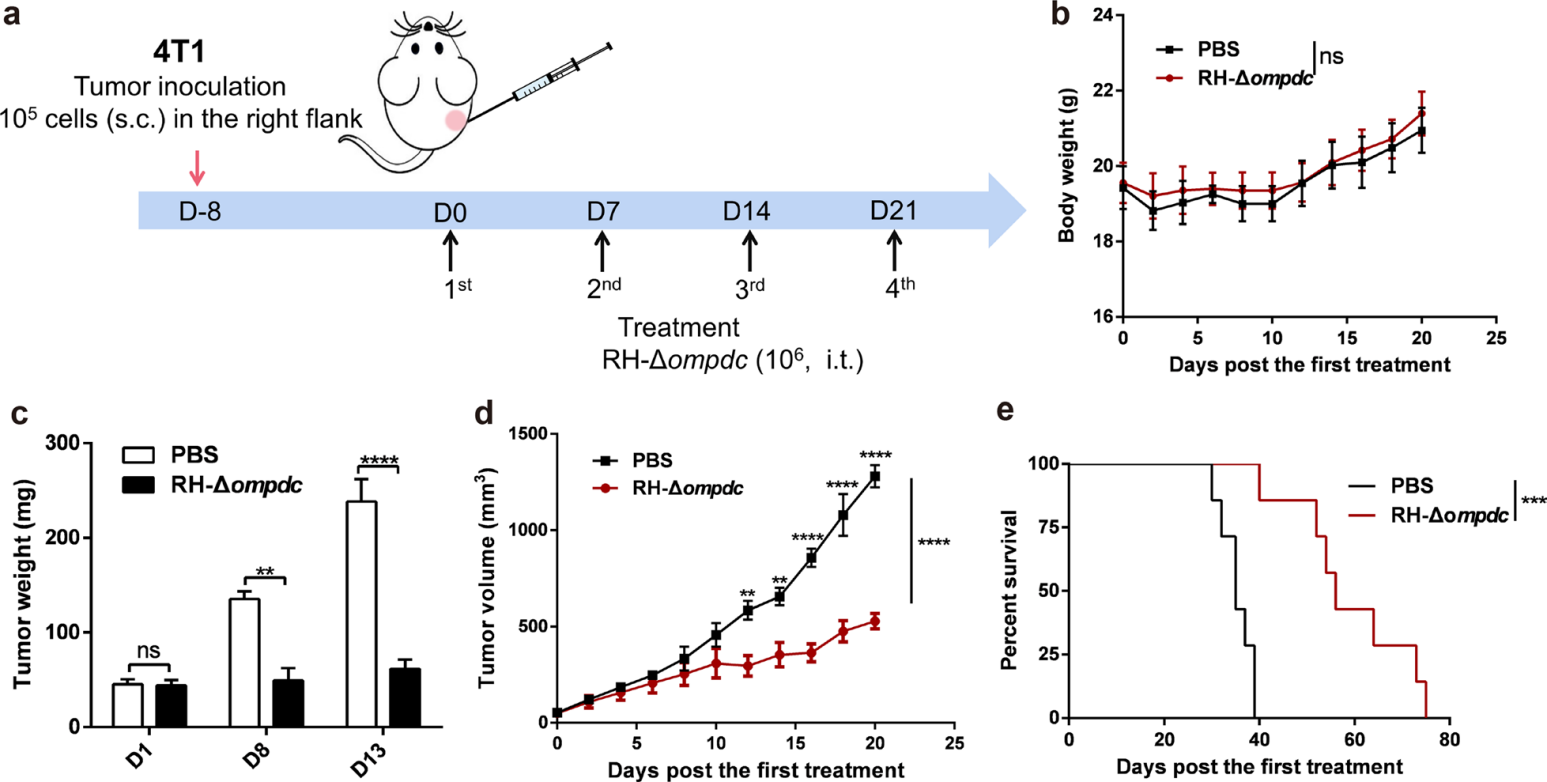
RH-Δ*ompdc* treatment reduced 4T1 tumor burden and improved survival of the tumor-bearing mice.** a** Schedule of RH-Δ*ompdc* treatment for the tumor-bearing mice. **b**–**e** Mice weight (**b**; *n* = 5), tumor weight (**c**; *n* = 4), tumor volume (**d**;* n* = 5) and survival curves (**e**; *n* = 7) of the established 4T1 tumor-bearing mice treated with RH-Δ*ompdc* tachyzoites or PBS. Results are representative of three independent experiments. The difference between groups was analyzed by two-way ANOVA with Tukey’s test (**b**–**d**), and survival data were analyzed by survival curves: log-rank (Mantel–Cox) test (**e**) Asterisks indicate significant difference at **P < 0.01, ***P < 0.001, **** *P* < 0.0001; ns, not significant. Abbreviations: i.t., intratumoral injection; s.c., subcutaneous injection

The 4T1 breast carcinoma is derived from BALB/c mice and shows many common characteristics with the naturally occurring human TNBC. Therefore, this carcinoma is widely used as a tumor model to evaluate the therapeutic approaches for human mammary carcinoma. We assessed the efficacy of RH-Δ*ompdc* for treating 4T1 murine breast cancer. RH-Δ*ompdc* tachyzoites were inoculated i.t. at a dose of 10^6^ tachyzoites per injection. The treatments were administered every 7 days for a total of four times (Fig. 5a). The RH-Δ*ompdc* treatment was well-tolerated by mice, with no significant body weight loss in the treatment and control groups throughout the observation period (Fig. 5b). However, the RH-Δ*ompdc* treatment remarkably inhibited tumor growth. The weight and volume of the tumors significantly decreased in the RH-Δ*ompdc* treatment group compared to the PBS treatment group (Fig. 5c, d). At 20 days after the initial treatment, tumor volumes in the PBS treatment group were approximately threefold larger than those in the RH-Δ*ompdc* treatment group (Fig. 5d). The RH-Δ*ompdc* treatment also significantly improved the survival of the tumor-bearing mice, with a median survival of 56 days, compared to only 36 days in the PBS treatment group (Fig. 5e). These results indicated that anti-tumor immune responses were induced after RH-Δ*ompdc *in situ inoculation into 4T1 tumors, leading to the inhibition of tumor growth and improved survival of the 4T1 tumor-bearing mice.

### RH-Δ*ompdc* treatment inhibited 4T1 cell lung metastasis

**Fig. 6:**
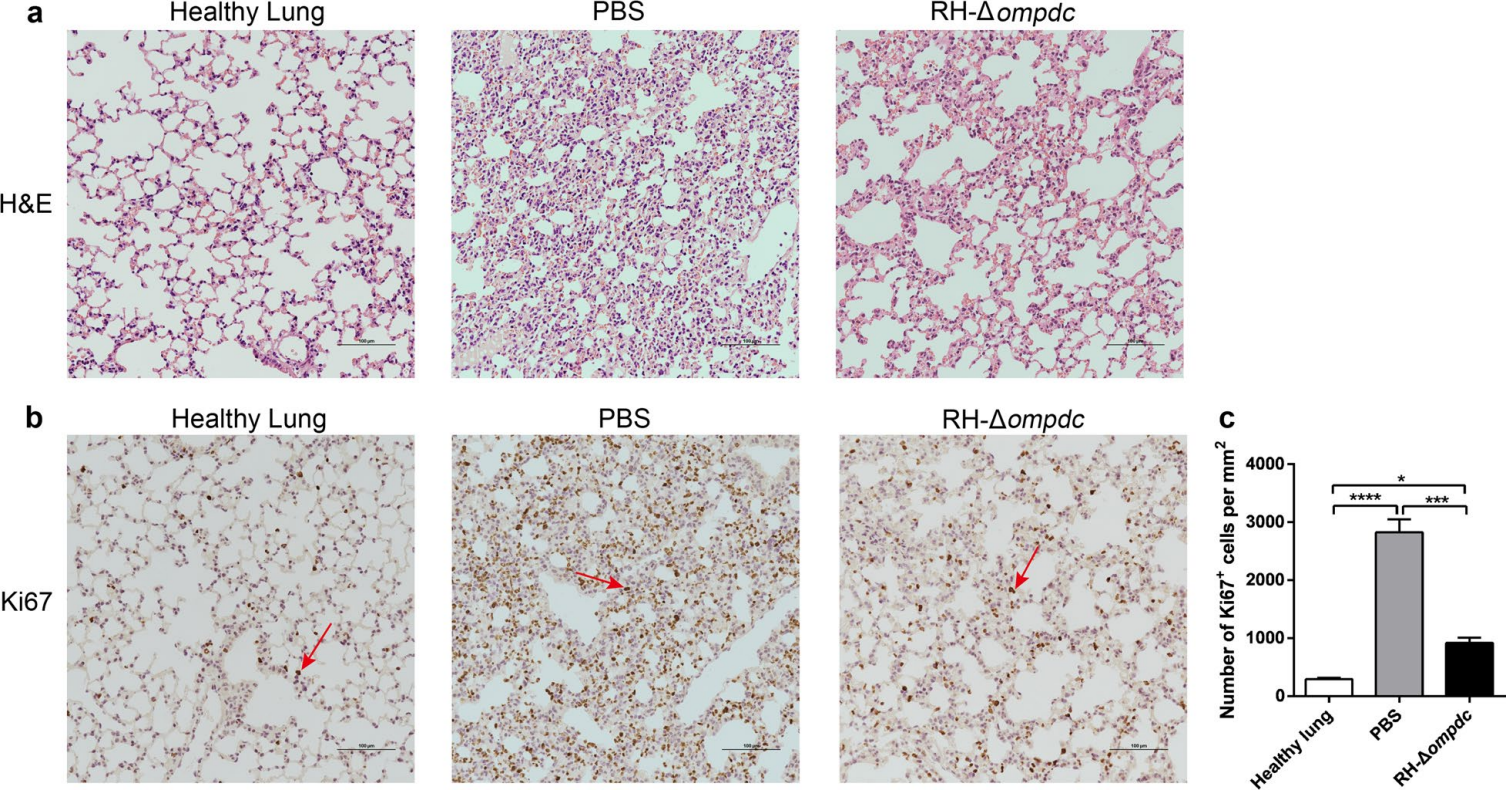
RH-Δ*ompdc* treatment inhibited 4T1 cell lung metastasis. The lungs of healthy mice and tumor-bearing mice (*n* = 3) receiving PBS or RH-Δ*ompdc* treatment were harvested 13 days after the first treatment, and the lung sections were stained with H&E or a Ki67 antibody. **a** Representative lung sections subjected to H&E staining. **b** Representative lung sections of the immunohistochemical staining with the Ki67 antibody. Ki67-positive cells are indicated with red arrows (scale bar: 100 μm). **c** Abundance of dividing cells (Ki67-positive cells/mm^2^) in the lung sections. Results are representative of three independent experiments. Data were analyzed by one-way ANOVA with Tukey’s test. Asterisks indicate significant difference at *P < 0.05, **P < 0.001, ****P < 0.0001. *A&E* Hematoxylin and eosin

4T1 metastatic disease spontaneously develops in the lungs of mice from the primary tumor as early as 8 days post inoculation, with increasing tumor burden at later time points [46]. In 4T1 tumor-bearing mice, lung metastasis is characterized by the infiltration of myeloid-derived suppressor cells and granulocytes [47]. The RH-Δ*ompdc* treatment of 4T1 tumors was carried out as described in the Methods section. The lungs of the mice were harvested on day 13 after the first treatment, stained with H&E and observed under a light microscope. Fewer infiltrating myeloid cells and larger alveoli spaces were found in the lung tissue of RH-Δ*ompdc* treatment group compared to that of the PBS group (Fig. 6a). The results indicated that extensive metastatic tumors had formed in the lungs of PBS-treated mice and that the lung architecture of RH-Δ*ompdc*-treated mice was similar to that of the healthy mice (Fig. 6a). The Ki67 protein, a non-histone nuclear protein, is widely used as a proliferation marker for tumor cells. Due to the lack of specific markers for metastatic 4T1 tumor cells, we detected the 4T1 cells in lungs by immunohistochemical staining with the Ki67 antibody. The results showed that the number of Ki67-positive cells was significantly reduced in the RH-Δ*ompdc* treatment group compared to the PBS group (Fig. 6b). The quantification of stained sections also showed a significant reduction in the number of Ki67-positive cells in the lung tissues of the RH-Δ*ompdc* treatment group compared to the PBS group (Fig. 6c). In summary, the RH-Δ*ompdc* treatment was able to effectively inhibit the 4T1 lung metastasis.

### RH-Δ*ompdc* treatment increased the IL-12 and IFN-γ level in both the serum and tumor microenvironment (TME)

**Fig. 7:**
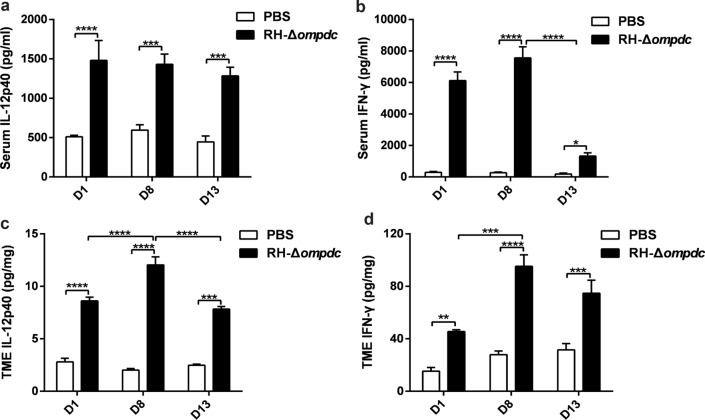
RH-Δ*ompdc* treatment increased the levels of IL-12 and IFN-γ in both the serum and TME. The 4T1 tumor-bearing mice model was established as illustrated in Fig. 5a, and the levels of the cytokines IL-12 and IFN-γ in the serum and TME were determined by ELISA at 1, 8, and 13 days after the first treatment. **a**, **b** Levels of IL-12 (**a**) and IFN-γ (**b**) in the serum were detected (*n* = 4). **c**, **d** Levels of IL-12 (**c**) and IFN-γ (**d**) in the TME were detected (*n* = 3). Results are representative of three independent experiments. Data were analyzed by two-way ANOVA with Tukey’s test. Asterisks indicate significant difference at *P < 0.05, **P < 0.01, ***P < 0.001, and ****P < 0.0001

Cytokines, such as the proinflammatory cytokines IL-12 and IFN-γ, play crucial roles in inhibiting the progression of breast cancer, so cytokine analysis of the serum and TME may provide important clues about the immunomodulatory effects of RH-Δ*ompdc* treatment. Following RH-Δ*ompdc *in situ inoculation to the 4T1 tumor, we determined the cytokine levels by ELISA in serum and homogenized tumor lysate collected 1, 8, and 13 days after the first treatment. The levels of IL-12 and IFN-γ in the RH-Δ*ompdc* treatment group were significantly upregulated in both the serum and TME, as compared to the PBS group (Fig. 7). However, the levels of IL-12 and IFN-γ were decreased in both the serum and TME on day 13 after the first treatment (Fig. 7). Overall, the higher expression levels of IL-12 and IFN-γ induced by the RH-Δ*ompdc* treatment implied that this uracil auxotroph *T. gondii* may activate immune cells and induce anti-tumor immune responses.

## Discussion

**Fig. 8:**
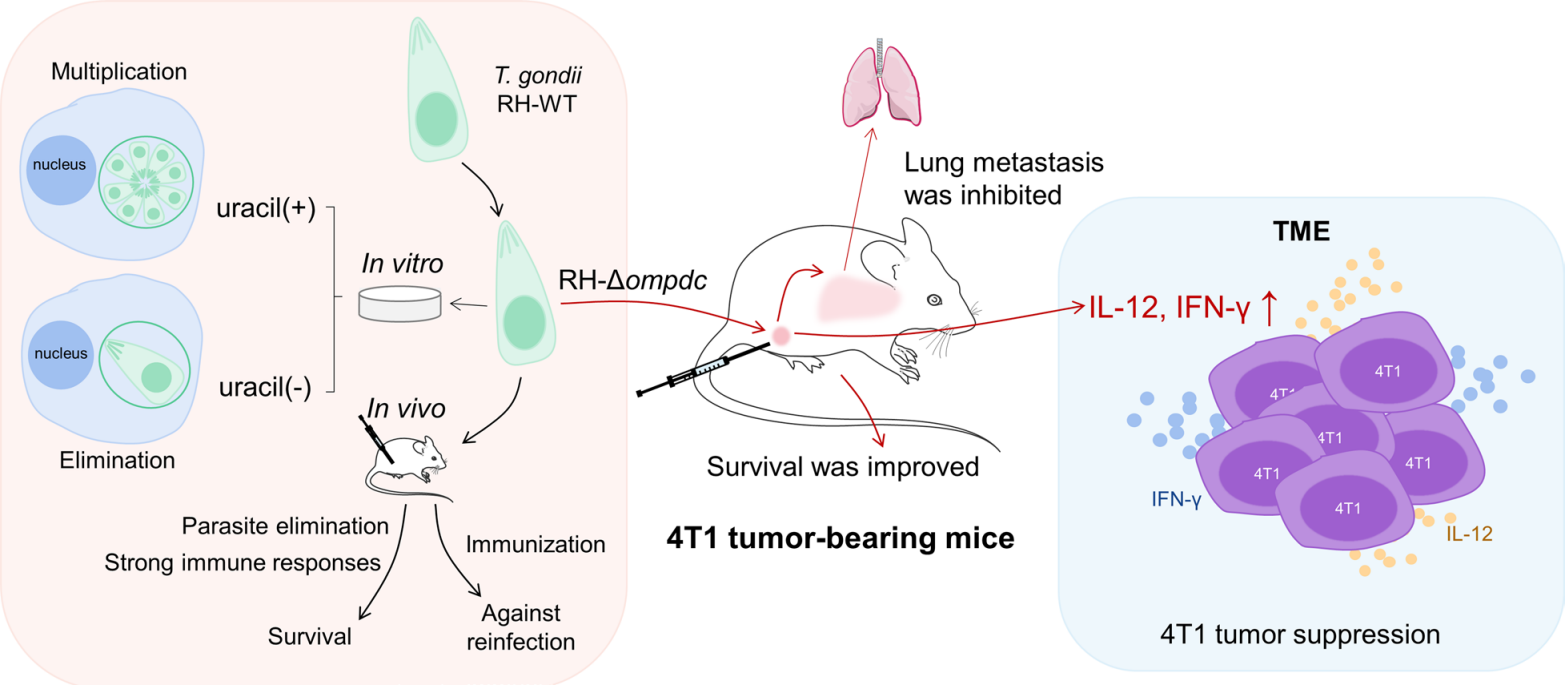
The possible anti-tumor and anti-infection immunity stimulated by RH-Δ*ompdc*. RH-Δ*ompdc* mutant was constructed with the CRISPR/Cas9 technology. RH-Δ*ompdc* could multiply normally in the medium supplemented with uracil, but it was cleared without uracil and in vivo. RH-Δ*ompdc* could induce host immune responses, both in vitro and in vivo, by promoting the expression of inflammatory cytokines, thus protecting the mice from reinfection by the RH strain. The RH-Δ*ompdc* tachyzoites in situ inoculation to 4T1 tumors inhibited the tumor growth, promoted the survival and reduced the lung metastasis of the tumor-bearing mice. After the RH-Δ*ompdc* treatment, the secretion of Th1 cytokines such as IL-12 and IFN-γ were significantly elevated in both the serum and TME, so the immune cells may be activated to recognize and kill tumor cells. Our study indicated a highly potential biotherapy for RH-Δ*ompdc* being used in cancer treatment

*Toxoplasma gondii* is frequently used as a model pathogen for the studies of Th1 cell-mediated immunity in intracellular infections. It has been reported that live avirulent parasites are more capable of inhibiting the growth of mice melanoma, ovarian, and pancreatic cancer than dead parasites, which suggests that the active invasion of the parasites is required for the induction of strong immune responses [34–36]. In our study, we constructed the uracil auxotroph *T. gondii* strain RH-Δ*ompdc*, and its virulence and the potential to be used in cancer biotherapy were evaluated. We found that RH-Δ*ompdc* proliferation was blocked in the absence of uracil in vitro, which was consistent with reports by other researchers [40, 41]. RH-Δ*ompdc* tachyzoites could also be eliminated in mice, suggesting that they are avirulent and safe to the hosts (Fig. 8). During *T. gondii* infection, the early production of IL-12 is driven by inflammatory monocytes [48, 49]. It is mainly active CD8α^+^ dendritic cells (DCs) [50], rather than neutrophils, that contribute to the production of IL-12 [51]. IL-12 signaling is a major pathway that leads to the secretion of IFN-γ by NK and T cells [52, 53], and IFN-γ is the major mediator of resistance against *T. gondii* in hosts [54, 55]. In our research, although proliferation was inhibited, RH-Δ*ompdc* tachyzoites still maintained the ability to induce immune responses as its wild-type strain, which significantly increased the expression of proinflammatory cytokines such as IL-12 and IFN-γ. Nevertheless, the immune responses tended to return to normal as the infection continued. Five days after infection with 10^6^ RH-Δ*ompdc* tachyzoites, the parasites in mice peritoneal fluids were almost eliminated, and the levels of IL-12 and IFN-γ had accordingly decreased, which indicated that the effect of a one-time treatment to the 4T1 tumor with RH-Δ*ompdc* may be not enough. Therefore, the tumor-bearing mice were treated every 7 days for a total of four times in our experiments. Cytokines and immune cells constitute a complex regulatory and effector network during disease processes; they are important components for host anti-infection and anti-tumor immunity [8, 56]. We therefore focused on the expression of IL-12 and IFN-γ, which are associated with breast cancer and positively influence anti-tumor immune responses.

As the most common aggressive breast cancer, TNBC remains difficult to control, despite a large number of therapeutic approaches having been evaluated. Nowadays, traditional treatments of breast cancer with surgery, radiation therapy and chemotherapy are the main therapeutic options in clinical practice [2, 5, 6]. However, chemotherapy and many of the novel immunotherapy strategies are associated with the development of complications. Therefore, it is urgent to search for other safe and effective therapeutic strategies targeting TNBC. The *T. gondii* uracil auxotroph Δ*cps* has shown strong anti-tumor activity in some tumor models, and the tumor growth inhibition of *T. gondii* treatment relies on CD8^+^ T cell activation [33–37]. Our results provide striking evidence of the distinct anti-tumor efficacy of RH-Δ*ompdc *in situ inoculation against 4T1 mouse breast tumors. RH-Δ*ompdc* treatment notably inhibited tumor growth, reduced lung metastasis and improved the survival of the tumor-bearing mice (Fig. 8). However, due to its resistance to pyrimethamine, the RH-Δ*ompdc* strain can only be used for experimental biotherapy.

Cytokine profiling indicated that secretion of IL-12 and IFN-γ significantly increased in the RH-Δ*ompdc* treatment group in both the serum and TME of tumor-bearing mice, suggesting vital roles for IL-12 and IFN-γ (Fig. 8). IL-12 and IFN-γ administration can inhibit the growth of 4T1 tumors [57–60]. IL-12 is mainly secreted by activated antigen-presenting cells, and it links innate immune responses with adaptive immune responses [61]. Additionally, in tumor immunotherapy, IL-12 therapy induces Th1 cell differentiation [62], increases the infiltration of NK and CD8^+^ T cells to tumor sites [63] and triggers an anti-angiogenic program to exert anti-tumor activity [64, 65]. Based on the higher detected levels of IL-12 and IFN-γ in our study, RH-Δ*ompdc* treatment may enhance anti-tumor immune responses. In the progression of anti-tumor immunity, NK cells, Th1 cells, and cytotoxic T lymphocytes (CTLs) are the main effector cells. IFN-γ is mainly secreted by NK and NKT cells in innate immunity and by Th1 or CTL cells in adaptive immunity [66]. IFN-γ plays a key role in the activation of cellular immunity and anti-tumor immune responses. IFN-γ signaling directly activates apoptotic processes and induces the apoptosis of cancer cells [67]. IFN-γ also inhibits angiogenesis in tumor tissue [68] and stimulates the activity of M1 macrophages to inhibit tumor progression [69]. The presence of these cytokines and immune cells in the TME has been shown to be associated with a good prognosis for many types of solid tumors, and abundant immunosuppressive cells, such as Tregs, MDSCs and TAMs, can achieve immune evasion and the tolerance of tumors [70, 71]. It is worth noting that Tregs are FoxP3^+^ CD4^+^ T cells that regulate immune tolerance and suppress dendritic cell (DC) maturation and CD8^+^ T cell recruitment to the TME [72]. MDSCs play an important role in inhibiting the activation of T cells, NK cells, and other immune cells [73]. TAMs usually display an anti-inflammatory M2-like phenotype and are frequently associated with tumor growth and metastasis [74]; they also suppress CD8^+^ T cell recruitment to the TME [75].

RH-Δ*ompdc* treatment increased the expression of IL-12 and IFN-γ, which may inhibit tumor angiogenesis, activate APCs and induce infiltrating T cells in the TME, thus reversing tumor immunosuppression to suppress 4T1 breast tumor growth and lung metastasis, and even induce systemic anti-tumor immunity. Further research is needed to reveal the anti-tumor mechanisms driven by IL-12 and IFN-γ in the 4T1 tumor model, and the activated DCs, NK, CD4^+^ and CD8^+^ T cells, as well as immunosuppressive cells in the TME. Furthermore, combination strategies may magnify anti-tumor immune responses and therapeutic effects [76, 77]. Therapies combining RH-Δ*ompdc* with other anti-tumor agents may achieve better therapeutic effects. RH-Δ*ompdc* may be a highly potential vaccine for treating malignancies, and the underlying anti-tumor mechanism is worth investigating.

## Conclusions

In summary, our study demonstrated that the RH-Δ*ompdc* mutant can grow normally in vitro with uracil supplement, but is unable to propagate without uracil addition and in vivo. Therefore, it is avirulent to the hosts. Inoculation of RH-Δ*ompdc *in situ resulted in anti-tumor efficacy against 4T1 tumor growth and lung metastasis. The RH-Δ*ompdc* infection increased the secretion of the Th1 cytokines IL-12 and IFN-γ, which may activate immune cells to recognize and kill tumor cells. However, mechanistic experiments still need to be performed to determine the anti-tumor mechanisms that are driven by IL12 and IFN-γ in the 4T1 tumor model. Our study indicates that RH-Δ*ompdc* therapy has high potential as a biotherapy for the treatment of breast cancer.

## Supplementary Information

**Additional file 1: Figure S1.** The primers used for construction and identification of the RH-Δ_ompdc_ mutant.

**Additional file 2: Table S1.** The primers used for qRT-PCR.

## Data Availability

The datasets supporting the findings of this article are included within the article.

## Abbreviations

ELISA: Enzyme-linked immunosorbent assay
GAPDH: Glyceraldehyde 3-phosphate dehydrogenase
HFF: Human foreskin fibroblast
IFN: Interferon
IL: Interleukin
iNOS: Inducible nitric oxide synthase
OMPDC: Orotidine 5′-monophosphate decarboxylase
qRT-PCR: Quantitative reverse transcription PCR
TME: Tumor microenvironment
TNBC: Triple-negative breast cancer
TNF-α: Tumor necrosis factor-α
