# Supplementary figures and images for: A uracil auxotroph Toxoplasma gondii exerting immunomodulation to inhibit breast cancer growth and metastasis
Source: Parasit Vectors. 2021 Dec 11;14:601. doi: 10.1186/s13071-021-05032-6 (PMC8665513; doi:10.1186/s13071-021-05032-6)

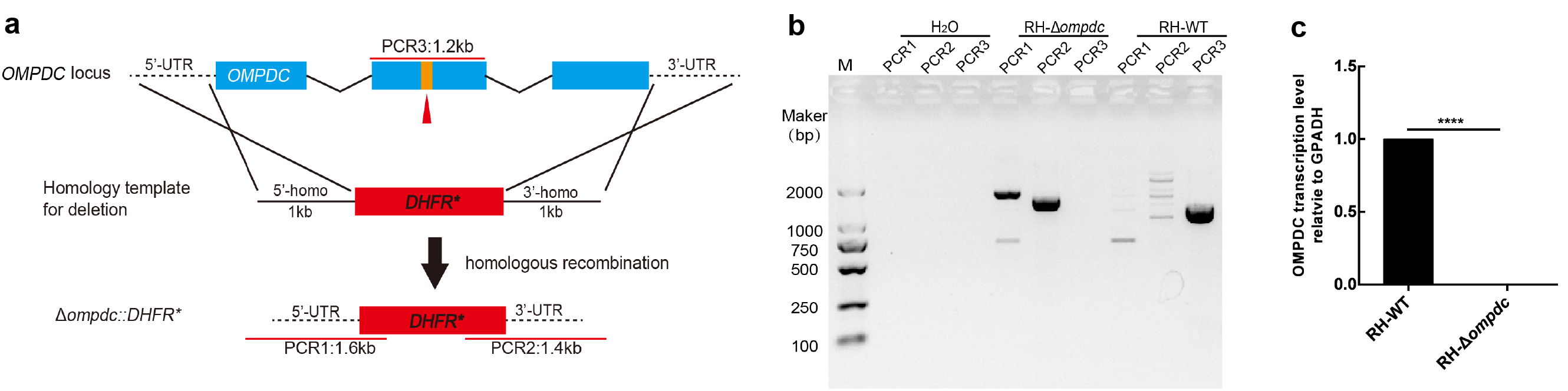

Supplement: Supplementary file 1 — Additional file 1: Figure S1. The primers used for construction and identification of the RH-Δompdc mutant. [file 13071_2021_5032_MOESM1_ESM.tif]
