# Supplementary material for: A uracil auxotroph Toxoplasma gondii exerting immunomodulation to inhibit breast cancer growth and metastasis
Source: Parasit Vectors. 2021 Dec 11;14:601. doi: 10.1186/s13071-021-05032-6 (PMC8665513; doi:10.1186/s13071-021-05032-6)
Supplement: Supplementary file 2 — Additional file 2: Table S1. The primers used for qRT-PCR. [file 13071_2021_5032_MOESM2_ESM.docx]

**Additional file 2**

**Additional file 2: Table S1.** The primers used for construction and identification the RH-Δ*ompdc* mutant.

| **Target** | **Forward primers (5’-3’)** | **Reverse primers (5’-3’)** |
| --- | --- | --- |
| OMPDC-5'homo | CGGCTTAAGTCAGTGTCGCGGG  CTG | CGCGGATCCTTAATTAACTTATTTGACAGTTC |
| OMPDC-3'homo | ATAAGAATGCGGCCGCATTTAA  ATCGTGCTGAAAGCGAAAC | TCCGGGCCCATTAAAAAAAGTACCTTGTC |
| DHFR | CCTTAATTAACTGTAAATCCCGT  GAGTCGTCC | CGATTTAAATACGTAGGAATTCATCCT |
| gRNA-OMPDC | GTTTCAGACCCTGCCAGTGAGTT  TTAGAGCTAGAAATAGC | AACTTGACATCCCCATTTAC |
| PCR1 | AGAAGTGGAAACCGAGTGTATG | GATGCCAAGGGCGTTATCT |
| PCR2 | TCCACTCGTGAATGCGTTATC | GGTGTTTCGTGACGTTCATTTC |
| PCR3 | GATTCCCGACGATGTTCCTATT | CCTCGCGAAGAGCTTCTATATC |
| RT-GAPDH | ATTTTGCTTGGGATTCGAGGA | TGCAGGGTAACGATCAAAAAATG |
| RT-OMPDC | GTCCAGGCTGCTGTGAATAC | CGACTGTGAGCAAGGAATCTAC |
| RT-B1 | GGAACTGCATCCGTTCATG | TCTTTAAAGCGTTCGTGGTC |

OMPDC: orotidine 5′-monophosphate decarboxylase; GAPDH: glyceraldehyde 3-phosphate dehydrogenase; DHFR: dihydrofolate reductase.

**Additional file 2: Table S2.** The primers used for qRT-PCR.

| **Target** | **Primers (5’-3’)**  **F:Foward R:Reverse** | **Product length (bp)** | **GenBank accession number** |
| --- | --- | --- | --- |
| GAPDH | F: CAACTTTGGCATTGTGGAAGG  R: ACACATTGGGGGTAGGAACAC | 224 | NM_001289726.1 |
| IL-12p40 | F: GATGTCACCTGCCCAACTG  R: TGGTTTGATGATGTCCCTGA | 120 | NM_001303244.1 |
| IL-6 | F: CCGGAGAGGAGACTTCACAG  R: CATTTCCACGATTTCCCAGA | 106 | NM_031168.2 |
| IL-1β | F: AACCTGCTGGTGTGTGACGTTC  R: CAGCACGAGGCTTTTTTGTTGT | 78 | NM_008361.4 |
| iNOS | F: CACCTTGGAGTTCACCCAGT  R: ACCACTCGTACTTGGGATGC | 170 | NM_010927.4 |
| TNF-α | F: ACGGCATGGATCTCAAAGAC  R: GTGGGTGAGGAGCACGTAGT | 116 | NM_013693.3 |

GAPDH: glyceraldehyde 3-phosphate dehydrogenase; IL-12: interleukin-12; IL-6: interleukin-6; IL-1β: interleukin-1β; iNOS: inducible nitric oxide synthase; TNF-α: tumor necrosis factor-α.
